# Viral Load Affects the Immune Response to HBV in Mice With Humanized Immune System and Liver

**DOI:** 10.1053/j.gastro.2017.08.034

**Published:** 2017-12

**Authors:** Mathilde Dusséaux, Guillemette Masse-Ranson, Sylvie Darche, James Ahodantin, Yan Li, Oriane Fiquet, Elodie Beaumont, Pierrick Moreau, Lise Rivière, Christine Neuveut, Patrick Soussan, Philippe Roingeard, Dina Kremsdorf, James P. Di Santo, Helene Strick-Marchand

**Affiliations:** 1 Innate Immunity Unit, Institut Pasteur, 75724 Paris, France; 2 INSERM U1223, Paris, France; 3 INSERM U1135, Faculté de Médecine, Université Pierre et Marie Curie Paris 6, Paris, France; 4 INSERM U966, Université François Rabelais and CHRU de Tours, Tours, France; 5 Unité des Hépacivirus et Immunité Innée, Institut Pasteur, 75724 Paris, France

**Keywords:** NK Cell, CXCL10, Mouse Model, IL10, HBe, cccDNA, covalently closed circular DNA, CHB, chronically HBV infected, ELISA, enzyme-Linked Immunosorbent Assay, ETV, entecavir, hAlb, human Albumin, HBcAg, hepatitis B core antigen, HBe, hepatitis B envelope, HBs, hepatitis B surface, HBsAg, hepatitis B surface antigen, HBV, hepatitis B virus, HCC, hepatocellular carcinoma, HIS, human immune system, HUHEP, human hepatocytes, IFN, interferon, LPC, liver progenitor cell, NK, natural killer, NUC, nucleos(t)ide analog, PD-L1, PD-1 ligand, PD-1, programmed death-1, qPCR, quantitative polymerase chain reaction, TLR, Toll-like receptor, TNF, tumor necrosis factor, TRAIL, TNF-related apoptosis-inducing ligand death receptor, wpi, week post-infection

## Abstract

**Background & Aims:**

Hepatitis B virus (HBV) infects hepatocytes, but the mechanisms of the immune response against the virus and how it affects disease progression are unclear.

**Methods:**

We performed studies with BALB/c *Rag2*^–/–^*Il2rg*^–/–^*Sirpa*^NOD^Alb-uPA^tg/tg^ mice, stably engrafted with human hepatocytes (HUHEP) with or without a human immune system (HIS). HUHEP and HIS-HUHEP mice were given an intraperitoneal injection of HBV. Mononuclear cells were isolated from spleen and liver for analysis by flow cytometry. Liver was analyzed by immunohistochemistry and mRNA levels were measured by quantitative reverse transcription polymerase chain reaction (PCR). Plasma levels of HBV DNA were quantified by PCR reaction, and antigen-specific antibodies were detected by immunocytochemistry of HBV-transfected BHK-21 cells.

**Results:**

Following HBV infection, a complete viral life cycle, with production of HBV DNA, hepatitis B e (HBe), core (HBc) and surface (HBs) antigens, and covalently closed circular DNA, was observed in HUHEP and HIS-HUHEP mice. HBV replicated unrestricted in HUHEP mice resulting in high viral titers without pathologic effects. In contrast, HBV-infected HIS-HUHEP mice developed chronic hepatitis with 10-fold lower titers and antigen-specific IgGs, (anti-HBs, anti-HBc), consistent with partial immune control. HBV-infected HIS-HUHEP livers contained infiltrating Kupffer cells, mature activated natural killer cells (CD69+), and PD-1+ effector memory T cells (CD45RO+). Reducing the viral inoculum resulted in more efficient immune control. Plasma from HBV-infected HIS-HUHEP mice had increased levels of inflammatory and immune-suppressive cytokines (C-X-C motif chemokine ligand 10 and interleukin 10), which correlated with populations of intrahepatic CD4+ T cells (CD45RO+PD-1+). Mice with high levels of viremia had HBV-infected liver progenitor cells. Giving the mice the nucleoside analogue entecavir reduced viral loads and decreased liver inflammation.

**Conclusion:**

In HIS-HUHEP mice, HBV infection completes a full life cycle and recapitulates some of the immunopathology observed in patients with chronic infection. Inoculation with different viral loads led to different immune responses and levels of virus control. We found HBV to infect liver progenitor cells, which could be involved in hepatocellular carcinogenesis. This is an important new system to study anti-HBV immune responses and screen for combination therapies against hepatotropic viruses.

See Covering the Cover synopsis on page 1459.

**Editor's Notes**

**Background and Context**

Although HBV infects hepatocytes, evasion and modulation of the immune response is key to the subsequent physiopathology and the mechanisms of disease progression are unclear.

**New Findings**

Chronic HBV immunopathology is modeled in a dually humanized mouse developing a complete viral life cycle and immune responses proportionally to the dose of viral inoculum.

**Limitations**

HBV-infected mice do not develop fibrosis.

**Impact**

Liver-specific immune responses are investigated during chronic HBV and innovative therapeutic approaches can be tested in this small animal model.

With over 350 million people infected by hepatitis B virus (HBV) and despite an efficient prophylactic vaccine, HBV prevalence is on the rise, constituting a major global health burden. The sequelae of HBV infection include acute and chronic hepatitis, which may lead to the development of fibrosis, cirrhosis, and hepatocellular carcinomas (HCC). The clinical course depends in part on the age of the host: most neonatally acquired infections develop into chronicity, whereas in adults they are mostly self-resolving.^1^ Current treatment strategies lead to viral suppression, but rarely establish a “functional cure” with HBsAg (hepatitis B surface antigen) loss. Furthermore, these life-long therapies do not eradicate the virus, nor do they efficiently train the immune response to control viremia, since viral rebound is frequent following discontinuation of treatment.^2^, ^3^ HBV is not directly cytopathic for hepatocytes, disease pathophysiology is conditioned by the ensuing anti-viral immune response.^4^ The breadth and scope of this response is essential for viral eradication; therefore, understanding the mechanisms that lead to viral clearance or persistence represents an essential goal.

Viral clearance is the result of a coordinated immune response initially mediated by Kupffer, natural killer (NK), and antigen-presenting cells, leading to robust and polyclonal CD4^+^ and CD8^+^ T-cell responses and the development of neutralizing anti-HBs antibodies that ensures protective immunity to functionally cured patients, despite the maintenance of covalently closed circular DNA (cccDNA).^4^, ^5^ In contrast, chronic viremia is associated with increased immunosuppressive cytokines (TGF-beta, IL-10) and regulatory T cells, combined with functional exhaustion of virus-specific T cells expressing programmed death-1 (PD-1), cytotoxic T lymphocyte-associated antigen-4 (CTLA-4) and tumor necrosis factor (TNF)-related apoptosis-inducing ligand death receptor (TRAIL-R2), targeting them for NK cell-mediated deletion.^4^, ^6^, ^7^, ^8^ In chronic HBV (CHB) patients, an increased risk of progression to cirrhosis and HCC has been correlated with the viral load; however, the mechanisms driving these virally induced pathologies are unclear.^9^, ^10^, ^11^

Deciphering the cross-talk between the immune system and HBV-infected hepatocytes has been hampered by the viruses’ restricted tropism to humans and chimpanzees. Although HBV transgenic or transduced mice, and HBV-infected human liver chimeric mice, are valuable for exploring the viral life cycle and testing antiviral compounds, they have limited immune responses and cannot assay immunotherapeutic approaches because significant differences between mice and humans predominate.^12^, ^13^

To establish an immunocompetent animal model for hepatotropic infections, several mouse models harboring both a humanized immune system and human hepatocytes have been recently described.^14^, ^15^, ^16^, ^17^, ^18^, ^19^ Although HBV infection of these models resulted in immune responses, low levels of liver chimerism limited viral output and the analysis of chronic pathophysiologic responses.^15^, ^19^

Previously, we established a dually humanized mouse model in BALB/c *Rag2*^*-/-*^*Il2rg*^*-/-*^*Sirpa*^*NOD*^ *Alb-uPA*^*tg/tg*^ recipients, stably engrafted with a humanized immune system and human hepatocytes (HIS-HUHEP mice).^18^ In this study, we demonstrate that HIS-HUHEP mice are susceptible to HBV, developing a full viral life cycle (HBeAg+, HBsAg+, cccDNA+) similar to CHB patients. By varying the viral inoculum in HIS-HUHEP mice, we found that distinct immune responses were obtained that led to different levels of viral control. Subsequent biomarker analysis revealed a cluster of effectors that may be useful to further dissect the complexity of HBV immune control. Nucleos(t)ide analog (NUC) treatment of chronically infected mice efficiently reduced viral loads, resulting in the resolution of liver inflammation.

## Materials and Methods

### Generation of Humanized Mice, HBV Infection, and NUC Treatment

HUHEP and HIS-HUHEP mice were established in BALB/c *Rag2*^-/-^*Il2rg*^-/-^*Sirpa*^NOD^uPA^tg/tg^ male and female mice as previously described.^18^ Mice with >100 μg/mL human Albumin (hAlbumin) and >10% hCD45+ cells in peripheral blood mononuclear cells (PBMC), including human T and B cells, were HBV infected intraperitoneally at 15 (±3) weeks with either 1x10^7^ or 1x10^9^ HBV genome equivalents (GE) purified from concentrated supernatants of the HepG2.2.15 stably producing cell line (HBV genotype D subtype ayw).^20^, ^21^ NUC-treated mice had been inoculated with 10^7^ HBV GE for 14 ±1 weeks prior to receiving Entecavir at 0.3 mg/kg/day either injected intraperitoneally or dispensed in the drinking water (Baraclude; Bristol-Myers Squibb, Princeton, NJ). Animals were housed in isolators under pathogen-free conditions with humane care. Experiments were approved by an institutional ethical committee at the Institut Pasteur (Paris, France) and validated by the French Ministry of Education and Research (MENESR #02162.02).

### Enzyme-Linked Immunosorbent Assay (ELISA) Analysis

Species-specific enzyme-linked immunosorbent assays (ELISA) of plasma samples for human albumin, human IgM, and human IgG were performed as previously described.^18^ M65 ELISA was performed according to manufacturer’s specifications (M65 EpiDeath ELISA; Peviva, Nacka, Sweden) on plasma samples pre- and post-HBV infection, or the equivalent time points for controls. M65 levels were normalized to hAlbumin levels for each time point. Human cytokines and chemokines were quantified in mouse plasma with the Human Cytokine Magnetic 25-plex Panel (catalog # LHC0009M; Life Technologies, Carlsbad, CA) according to manufacturer's specifications and analyzed with MAGPIX (Luminex, Austin, TX).

### Flow Cytometry Analysis

Mononuclear cells from blood, spleen, and liver were isolated as previously described.^18^ Cell labeling was performed with directly conjugated monoclonal antibodies against human CD3, CD4, CD8, CD14, CD16, CD33, CD45, CD45RA, CD45RO, CD56, CD69, HLA-DR, Nkp46, PD-1 (BD Biosciences, San Jose, CA; eBiosciences, San Diego, CA; Miltenyi Biotech, Bergisch Gladbach, Germany) according to standard techniques. Dead cells were excluded using Fixable viability dye (eBioscience). Intracellular labeling was performed as previously described^18^ (see Supplementary Materials and Methods). Acquisitions were performed using BD Fortessa and LSRII flow cytometers (Becton Dickinson, Franklin Lakes, NJ). Analysis was performed with FlowJo Version 8.8 (TreeStar, Ashland, OR) and Prism 6 (GraphPad, La Jolla, CA).

### Immunofluorescence Analysis

Liver cryostat sections were prepared as previously described^22^ and immunostained with antibodies against HBc (B0586 [Dako, Glostrup, Denmark] or C1-5 [Santa Cruz, Dallas, TX]), and human antigens: albumin (A0001 [Dako] or A80-129A [Bethyl]), CD3 (A0452 [Dako] or UCHT1 [eBiosciences]), CD45 (2D1 [BD Biosciences]), CD68 (KP1 [Dako]), CK7 (M7018 [Dako]) EpCAM (OP187 [Calbiochem, San Diego, CA]), PD-L1 (MAB1561 [R&D Systems]). Secondary antibodies were coupled to Alexa Fluor 488, Alexa Fluor 555, or Alexa Fluor 647 (Molecular Probes, Eugene, OR). Photomicrographs were taken with an Axioimager Apotome microscope (Zeiss, Oberkochen, Germany).

### Virological Measurements

HBV DNA was extracted from plasma and homogenized liver tissue using QIAamp DNA blood mini kit (Qiagen, Hilden, Germany). Plasmatic HBV DNA was quantified by quantitative polymerase chain reaction (qPCR) with a LightCycler system (Roche, Basel, Switzerland) as described by Brezillon et al.^21^ Intrahepatic HBV DNA was quantified by SYBR Green qPCR on an ABI PRISM 7900HT system (Applied Biosystems, Foster City, CA). For HBV cccDNA, DNA was pre-treated with 10 U plasmid-safe DNase (Epicenter, Madison, WI) for 1 hour at 37°C. HBV-specific primers were: 5′-GTTGCCCGTTTGTCCTCTAATTC-3′ and 5′-GGAGGGATACATAGAGGTTCCTTG-3′ for total HBV DNA; and 5′-GTGCACTTCGCTTCACCTCT-3′ and 5′-AGCTTGGAGGCTTGAACAGT-3′ for cccDNA amplification. Standard curves were generated from an HBV genome-containing plasmid (payw1.2), data was normalized to human cell numbers by qPCR amplification of the human IL8 promoter as described by Rivière et al.^23^ HBe and HBs antigens were quantified with a clinical test (DiaSorin, Saluggia, Italy).

### Antigen-Specific Antibody Responses

Clinical assays for HBsAb and HBcAb have detection thresholds well above the IgG antibody titers developed in HIS-HUHEP mice. HIS-HUHEP mouse plasma was assayed for antigen-specific antibodies by immunocytochemistry on transfected BHK-21 cells producing either HBs or HBc protein, or β-galactosidase as a control.^24^ BHK-21 cells were incubated with humanized mouse sera previously normalized to 80 μg/mL total human IgG followed by 2-fold serial dilutions (details provided in Supplementary Materials and Methods).

### Gene Expression Analysis by Quantitative Reverse-Transcription Polymerase Chain Reaction

cDNA was prepared from 200 ng of total liver RNA with 2 steps using Superscript III RT (Invitrogen) for reverse transcription followed by TaqMan PreAmp Master Mix (Applied Biosystems) for target amplification with all TaqMan gene expression assays pooled at 0.2x. Subsequently, specific gene expression was performed using BioMark 48.48 Dynamic Arrays (Fluidigm, San Francisco, CA) following manufacturer's protocols. For each sample, 3 independent experiments were analyzed with 2 housekeeping genes (HPRT and GAPDH). Human specificity of each gene expression assay was verified on C57BL/6 mouse liver samples.

### Statistical Analysis

Data sets were tested with 2-tailed unpaired Student *t* tests or Mann Whitney U tests, correlations were analyzed with Pearson's χ^2^ test using Prism version 6 (GraphPad Software, San Diego, CA). Significant *P* values are shown as: *P < .05, **P < .005, and ***P <.0005.

## Results

### Control of Viremia in Immunocompetent Humanized Mice Chronically Infected With HBV

**Figure 1:**
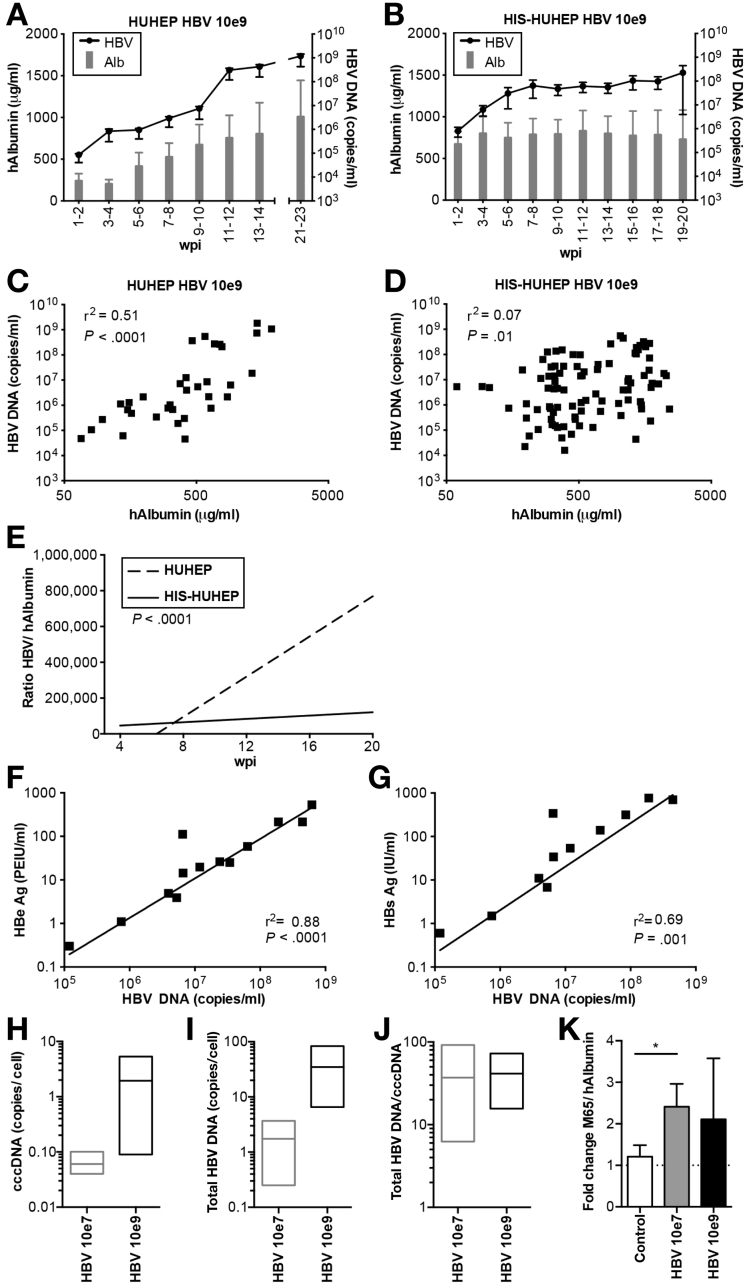
Viral progression is controlled in immunocompetent HIS-HUHEP mice. (*A* and *B*) HBV viremia (*black line*) and hAlbumin (*grey bars*) were measured in the plasma of HBV-infected HUHEP (*A*) and HIS-HUHEP (*B*) mice inoculated at 10e9. Means of HUHEP (n=4) and HIS-HUHEP (n=12) mice. (*C* and *D*) Analysis of viral load: for each plasma sample viremia was plotted against the hAlbumin concentration from HUHEP (*C*) (n=4) or HIS-HUHEP mice (*D*) (n=12). (*E*) Analysis of viral progression over time by linear regression analysis of the ratio of HBV DNA over hAlbumin concentration, plotted against time post infection, from HUHEP (*dotted line*) or HIS-HUHEP mice (*filled line*). The *P* value determines whether the slopes of each linear regression are significantly different from each other. (*F* and *G*) Plasma viral antigen loads (HBeAg and HBsAg) correlated with HBV viremia in HBV-infected HIS-HUHEP mice. (*H*, *I*, and *J*) Quantification of cccDNA and total HBV DNA in the liver of infected HIS-HUHEP mice. The ratio of total liver HBV DNA/cccDNA indicates the virus’ replicative activity (n=3). Bars show mininum to maximum, line at the mean. (*K*) Human hepatotoxicity (M65) was normalized to the degree of human liver chimerism (hAlbumin) per mouse. The fold change of M65/hAlbumin pre- and post-infection (19 ±4 wpi) are shown for each group (control n=8; HBV 10e7, n=7; HBV 10e9, n=4) Histograms show means and SEM. Statistical significance: Mann Whitney U test. Correlation analysis: r^2^ and *P* values calculated using 2-tailed Pearson's χ^2^ test.

**Supplementary Figure 1:**
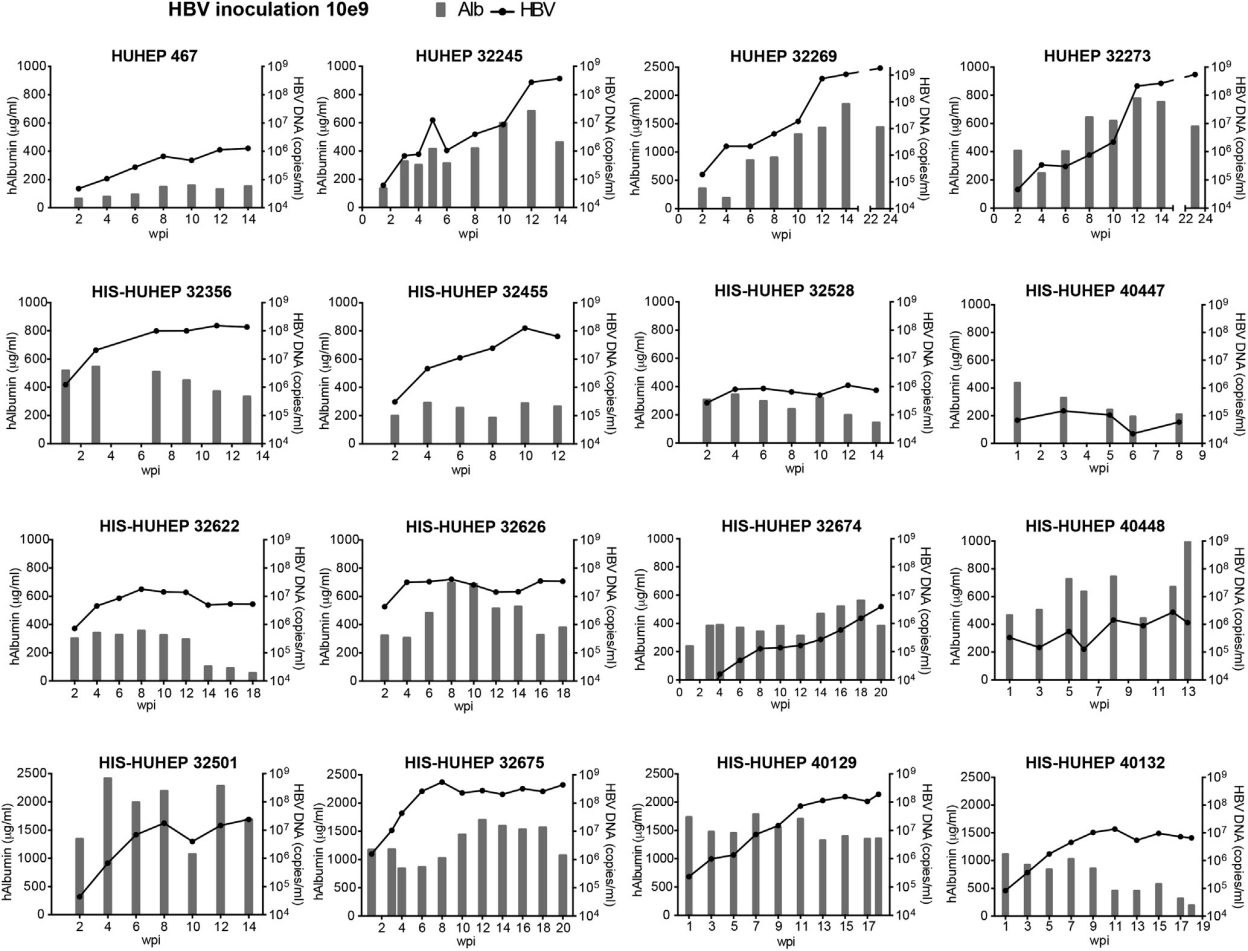
Longitudinal follow-up of HBV viremia (*black line*) and hAlbumin (*grey bars*) in the plasma of individual HUHEP and HIS-HUHEP mice inoculated with high-dose HBV 10e9.

**Supplementary Figure 2:**
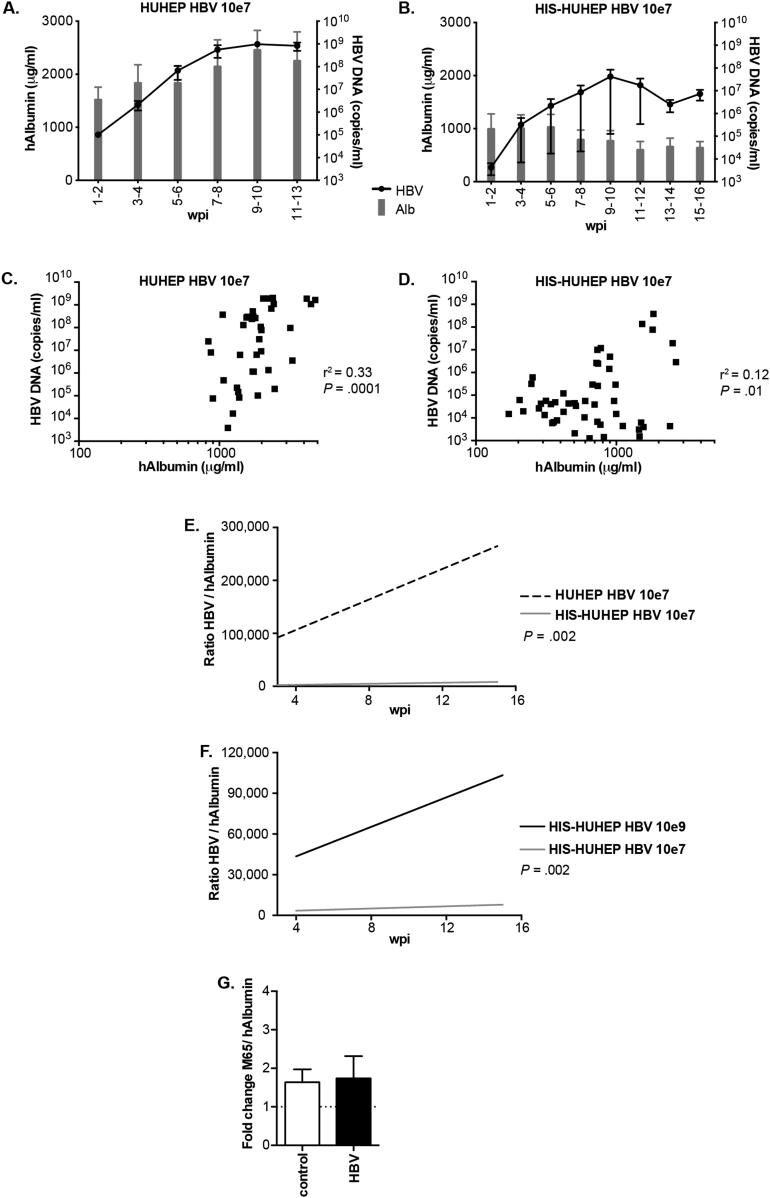
Follow-up of viral loads and liver chimerism in HIS-HUHEP mice inoculated at low-dose HBV 10e7. (*A* and *B*) HBV viremia (*black line*) and hAlbumin (*grey bars*) were measured longitudinally in the plasma of HBV-infected HUHEP (*A*) and HIS-HUHEP (*B*) mice. Graphs show the mean from 6 HUHEP and 6 HIS-HUHEP mice. (*C* and *D*) Correlative analysis of the viral load vs liver humanization: for each plasma sample viremia was plotted against hAlbumin concentration from HUHEP (*C*) (n=6) or HIS-HUHEP mice (*D*) (n=6). Correlation was evaluated using a 2-tailed Pearson's χ^2^ test. (*E* and *F*) Analysis of viral progression over time: linear regression analysis of the ratio of HBV viral load over hAlbumin concentration plotted against time post infection, from (*E*) HUHEP (*dotted line*) or HIS-HUHEP mice inoculated at 10e7 or (*F*) from HIS-HUHEP mice inoculated at 10e7 vs 10e9 (respectively, *grey* or *black filled line*). The *P* value determines whether the slopes are significantly different. (*G*) Fold change of M65/hAlbumin pre- and post-infection (17 ±6 wpi) in HUHEP mice (control n=4 HBV 10e7; and 10e9 n=6).

**Supplementary Figure 3:**
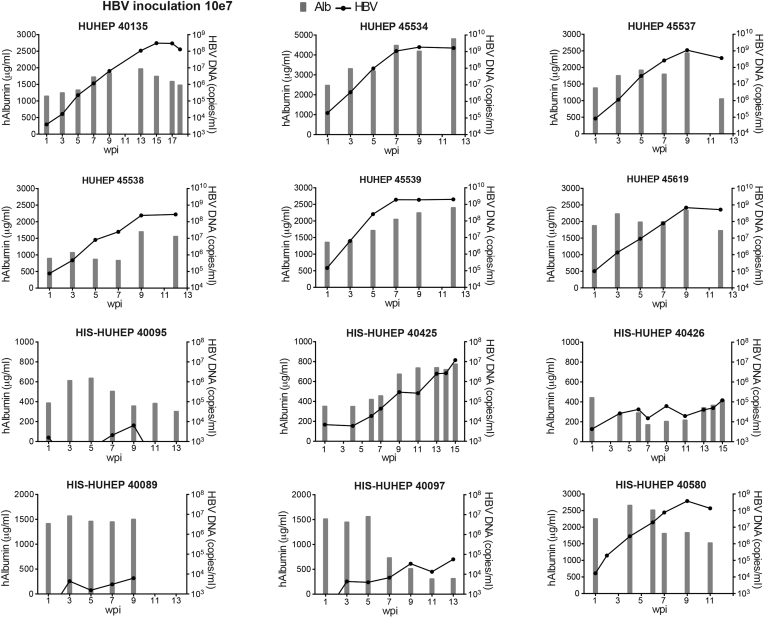
Longitudinal follow-up of HBV viremia (*black line*) and hAlbumin (*grey bars*) in the plasma of individual HUHEP and HIS-HUHEP mice inoculated with low-dose HBV 10e7.

We analyzed the outcome of HBV infection after ‘high dose’ inoculation (10^9^ HBV DNA copies) in HUHEP and HIS-HUHEP mice. Both models could be productively infected by HBV over the 4–5 month time course of the experiment (Figure 1, individual animals are shown in Supplementary Figure 1). While high levels of viral replication were evident in both models, viral progression was markedly different in the presence of human immune cells. Interestingly, viremia increased unchecked in HUHEP mice, whereas it stagnated in HIS-HUHEP mice, resulting in 10-fold lower viral titers in HIS-HUHEP compared with HUHEP mice (respectively, up to ≈10^8^ vs 10^9^ HBV DNA copies/mL); Figure 1*A* and 1*B*). We determined whether viral loads correlated with hAlbumin levels in HUHEP vs HIS-HUHEP models. In HUHEP mice, this correlation was significant (r^2^=0.51), whereas in immunocompetent humanized HIS-HUHEP mice there was no correlation (r^2^=0.07) (Figure 1*C* and 1*D*). Moreover, virus progression (ratio HBV DNA/hAlbumin) increased proportionally over time in the HUHEP mice (r^2^=0.72), but not in the HIS-HUHEP model (r^2^=0.03) (Figure 1*E*). The difference between HUHEP and HIS-HUHEP models was highly statistically significant (*P* < .0001) indicating that HBV infection was controlled in HIS-HUHEP mice, presumably through anti-HBV human immune responses. The cross-talk between infected hepatocytes and immune effectors may differ according to the viral load; chimpanzees inoculated with low or high doses of HBV had distinct intrahepatic immune responses, resulting in either a delayed or rapid viral clearance.^25^ We therefore analyzed HIS-HUHEP mice infected with a lower dose (10^7^ HBV DNA copies) to assess the impact of HBV load on viral progression. Following infection with the lower inoculum, viremia was globally reduced (maximum ≈10^7^ HBV DNA copies/mL), more efficiently controlled, and 1 HIS-HUHEP mouse cleared the infection (Supplementary Figures 2 and 3).

We characterized the viral life cycle in these HBV-infected humanized mouse models. Both HBeAg and HBsAg were within clinical ranges and correlated to HBV DNA viral titers (Figure 1*F* and 1*G*). cccDNA levels were higher in ‘high dose’ compared with ‘low dose’ inoculated mice (respectively, mean 1.9 vs 0.06 copies/cell; Figure 1*H* and 1*I*). However, the replicative activity (intrahepatic total HBV DNA/ cccDNA) was similar for both inocula (Figure 1*J*) and comparable to non-treated CHB patients.^26^

In our previous work, we found that human hepatocyte graft function, measured as plasma hAlbumin, generally increased over time in non-infected HUHEP and HIS-HUHEP mice.^18^ Interestingly, hAlbumin also increased in HBV-infected HUHEP mice, but this failed to occur in HBV-infected HIS-HUHEP mice (Figure 1, Supplementary Figure 1, Supplementary Figure 2, and 3). In uPA-based models, active tissue remodeling of the host, independently of the grafted cells, precludes use of ALT measurements (alanine aminotransferase) due to its lack of species specificity.^27^ Hepatotoxicity results in soluble and fragmented Cytokeratin-18 measurable in the plasma with a human-specific antibody M65 by ELISA.^28^ In non-infected HIS-HUHEP mice, M65 was stable over time, yet levels increased following HBV infection, indicating that human hepatocyte cytolysis was occurring (Figure 1*K*). No significant differences were observed in control or HBV-infected HUHEP mice (Supplementary Figure 2).

### Cellular Immune Responses in HIS-HUHEP Mice With Chronic HBV-Infection

**Figure 2:**
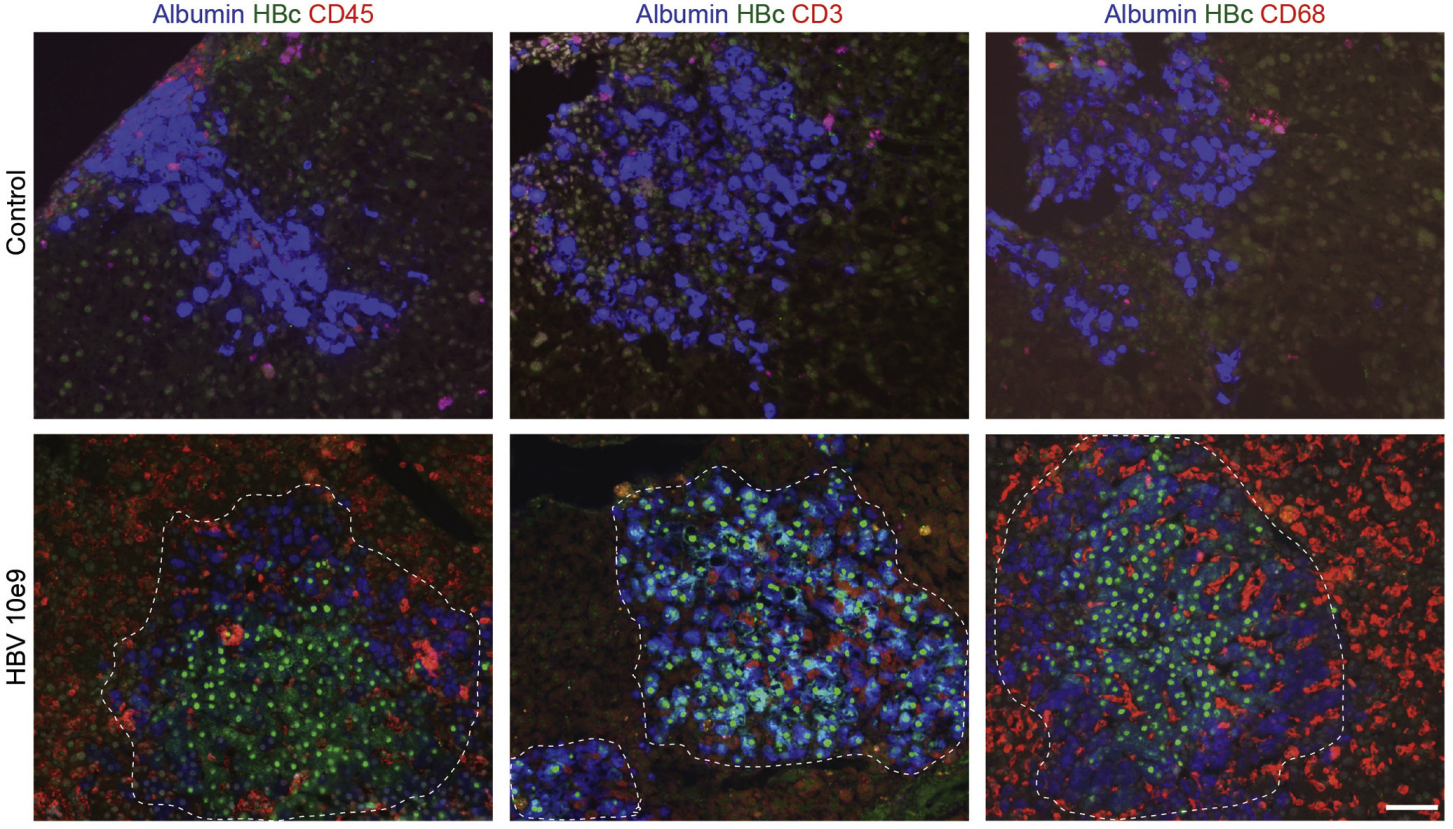
Human immune cells are recruited to the liver in chronically infected HIS-HUHEP mice. Immunofluorescence analysis of liver sections from control (*top panels*) and HBV-infected (*bottom panels*) mice co-stained for hAlbumin (*blue*) and HBc (*green*), with either hCD45 (*red*), or hCD3 (*red*), or hCD68 (*red*). DAPI-stained nuclei are shown in grey. Scale bar represents 100 μm.

**Figure 3:**
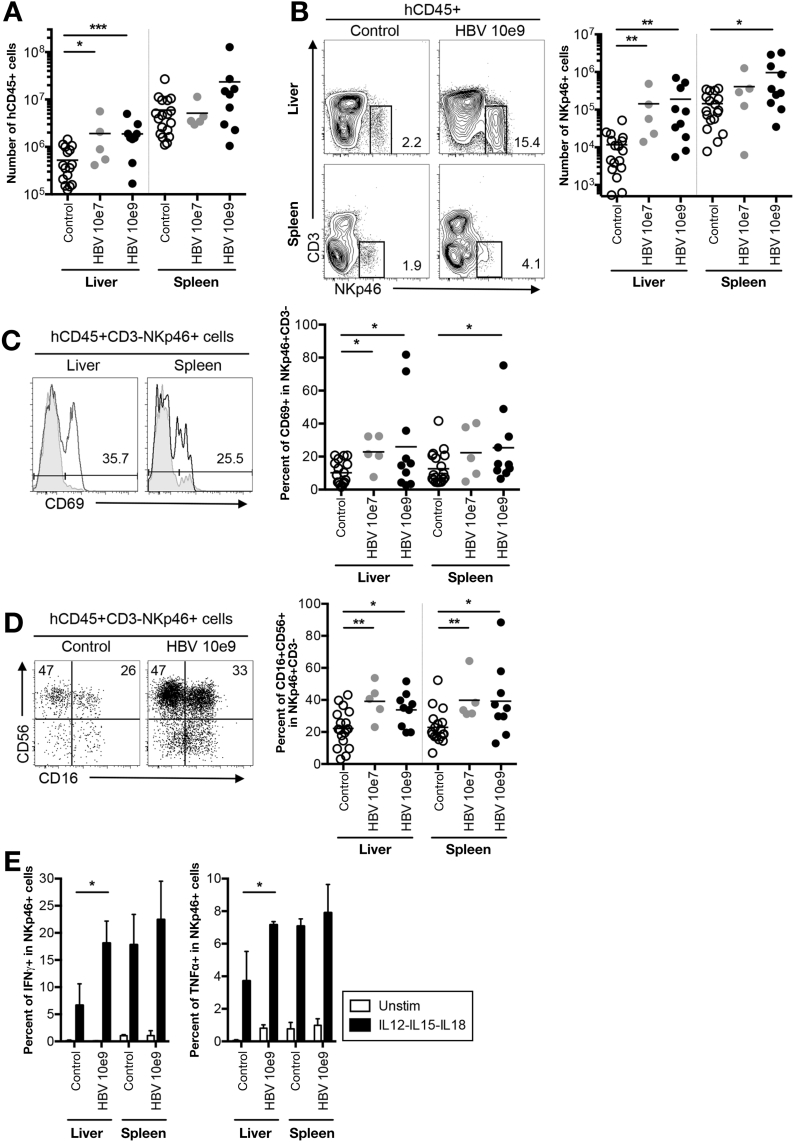
NK-cell mediated immune response in HBV-infected HIS-HUHEP mice. (*A*) Absolute numbers of total human leukocytes (hCD45+) and (*B*) NK cells (CD3^-^NKp46^+^) from the liver and spleen of control and HBV-infected mice. Representative FACS plots show the percent positive cells in each gate. (*C*) Frequency of CD69^+^ cells among NK cells in the liver and spleen from control (*grey filled line*) and HBV-infected (*black open line*) HIS-HUHEP mice. The percentage of positive cells from HBV-infected mice is shown. (*D*) Expression of CD56 and CD16 in NK cells from the liver of control or HBV-infected HIS-HUHEP mice. (*E*) Liver leukocytes or splenocytes were restimulated ex vivo overnight and analyzed by FACS for the expression of IFN-γ or TNF-α by NK cells. Histograms show the mean and SEM. In plots each dot represents a mouse, data obtained at 14–20 wpi. Statistical analysis in *A*–*D*: Mann Whitney U test, *E*: 2 way ANOVA.

**Supplementary Figure 4:**
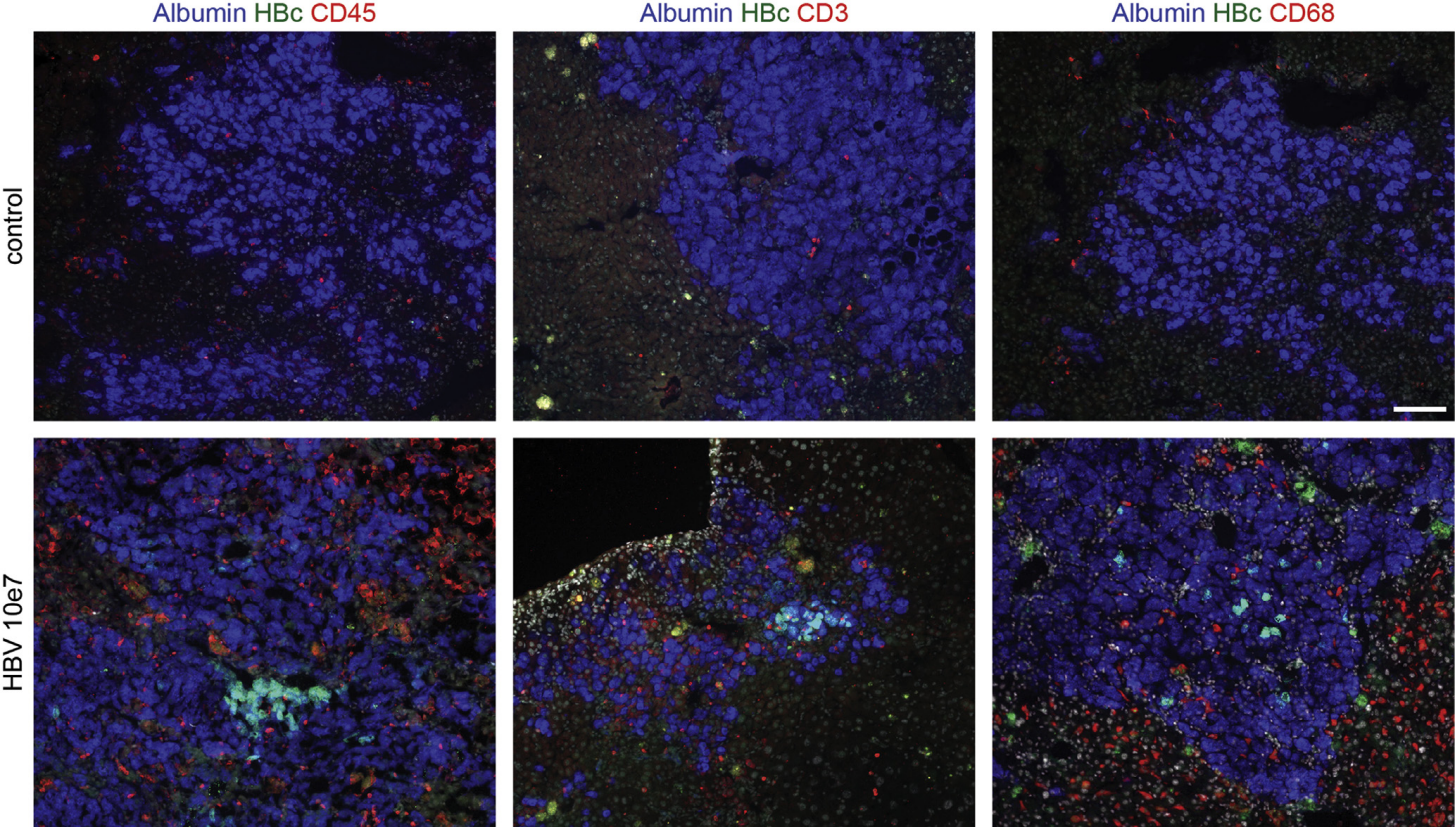
Immunofluorescence analysis of liver sections from HIS-HUHEP control or low-dose (HBV 10e7) infected mice co-stained for hAlbumin (*blue*) and HBc (*green*), with either hCD45 (*red*), or hCD3 (*red*), or hCD68 (*red*). DAPI-stained nuclei shown in *grey*. Scale bar represents 100 μm.

To assess human immune responses to HBV infection in situ, we immunostained liver sections of infected and control HIS-HUHEP mice. Most human hepatocytes were infected (hAlb^+^ HBcAg^+^) and strong inflammation (hCD45) composed of T cells (hCD3) and Kupffer cells (hCD68) was observed in the parenchyma of ‘high dose’ infected animals (Figure 2). Inflammatory foci of hCD45^+^ cells were grouped adjacent to HBcAg^+^ hepatocytes. Specifically, CD3^+^ T cells were concentrated within clusters of infected hepatocytes, whereas CD68^+^ Kupffer cells were present throughout the liver parenchyma and intertwined between infected hepatocytes (Figure 2). In ‘low dose’ infected mice, fewer hepatocytes were HBcAg^+^, and a diffuse recruitment of CD3^+^ T cells and CD68^+^ Kupffer cells to the liver was apparent (Supplementary Figure 4). Histologically, liver fibrosis was not observed and α-smooth muscle actin staining was normal (data not shown). Human CD45^+^ cellularity was significantly increased in the liver of HIS-HUHEP mice following HBV infection (Figure 3*A*). HBV-infected mice demonstrated a striking increase in the numbers of hepatic NK cells (hCD45^+^CD3^-^NKp46^+^) (for low and high dose, respectively, in the liver, 12x and 16x; in the spleen, 3x and 7x) (Figure 3*B*). More NK cells were activated (CD69^+^) and showed a mature phenotype (CD56^+^CD16^+^) in HBV-infected mice compared with controls (Figure 3*C* and 3*D*). Liver-derived NK cells from high-dose HBV-infected mice produced significantly more IFN-γ and TNF-α than from control mice after stimulation with IL-12, IL-15, and IL-18, suggesting heightened responsiveness subsequent to viral stimulation in vivo (Figure 3*E*). A similar trend was observed in the spleen, although the differences were not significant.

**Figure 4:**
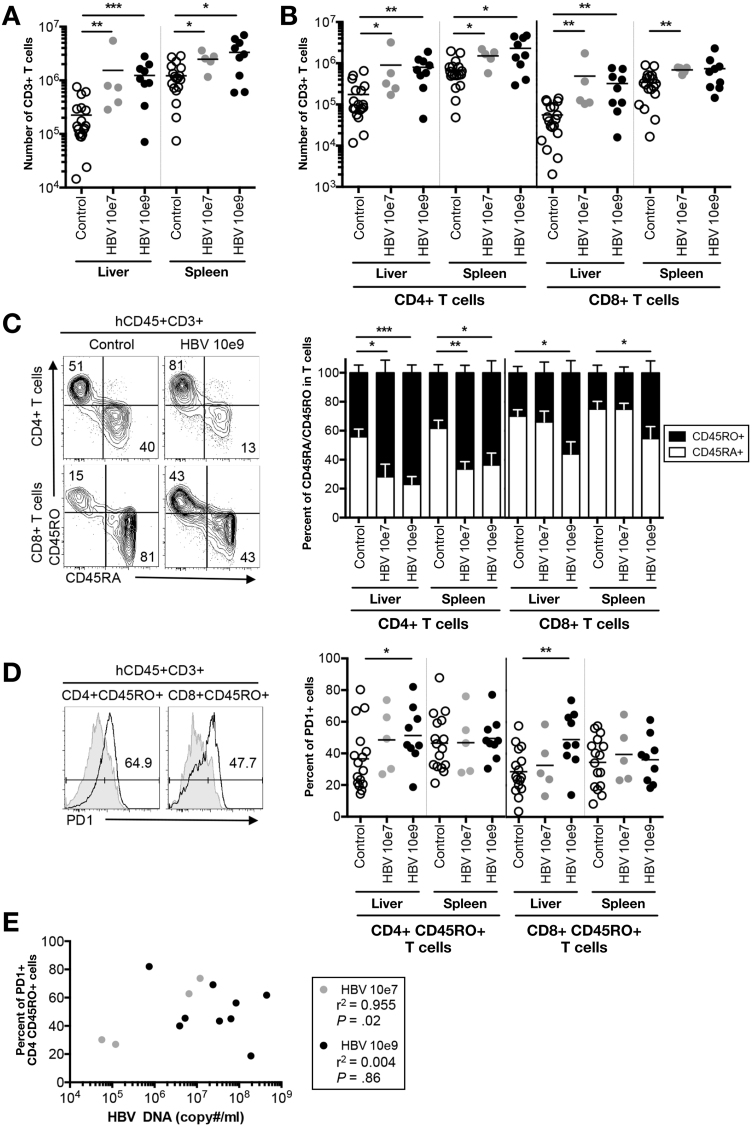
Characterization of human T-cell subsets during chronic HBV infection. (*A*) Total numbers of human T lymphocytes (hCD45^+^CD3^+^) and (*B*) CD4^+^ or CD8^+^ T cells isolated from the liver and spleen. (*C*) Representative FACS plots of CD4^+^ or CD8^+^ T cells from the liver of control or HBV-infected samples, histograms show normalized frequencies of naïve (CD45RA^+^ [*white bar*]) or memory (CD45RO^+^ [*black bar*]) cells among CD4^+^ or CD8^+^ T cells. Mean and SEM are shown. (*D*) Frequency of PD-1^+^ cells among the CD4^+^CD45RO^+^ or CD8^+^ CD45RO^+^ memory T cells from a representative HIS-HUHEP control (*grey filled line*) and HBV 10e9 inoculated (*black empty line*) mouse liver. Each dot represents a mouse, data obtained at 14–20 wpi. *A*–*D*: Mann Whiteny U test. (*E*) Analysis of an exhaustion marker (PD-1^+^) on intrahepatic CD4^+^CD45RO^+^ T cells as a function of viral load with Pearson’s correlation test.

**Supplementary Figure 5:**
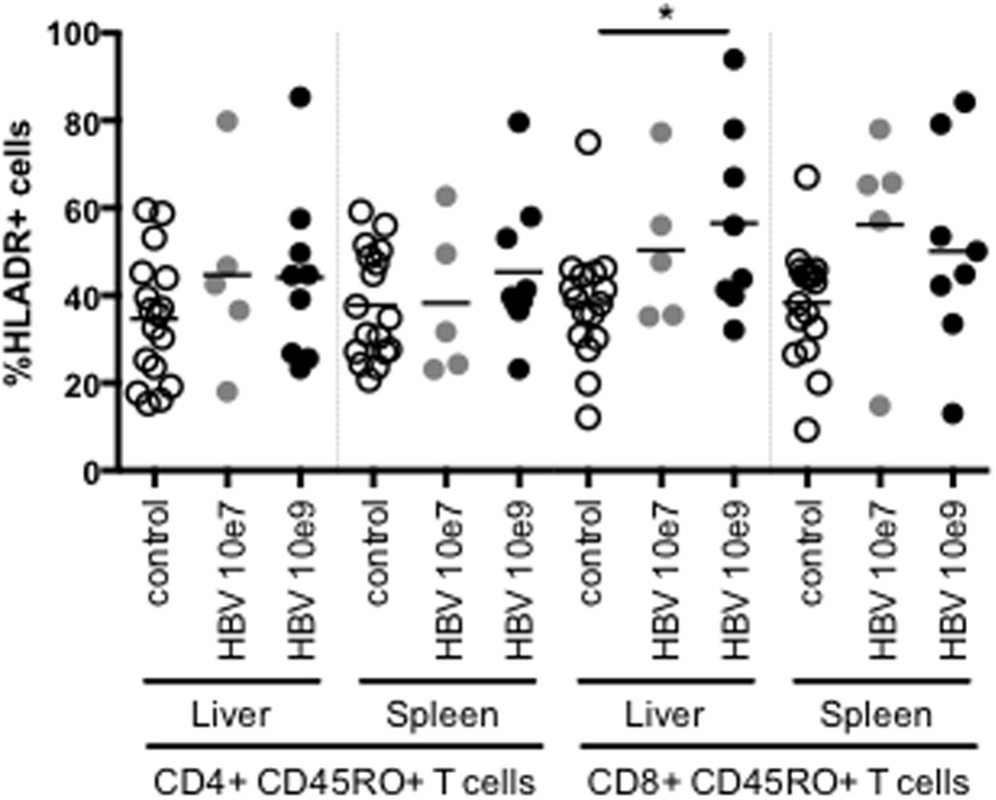
Characterization of human T-cell subsets in HIS-HUHEP–infected mice by FACS analysis. Percentage of HLA-DR–positive cells in memory CD4^+^CD45RO^+^ or CD8^+^CD45RO^+^ T cells from control or HBV-infected mice at low (HBV 10e7) or high (HBV 10e9) doses.

Persistence of viral antigens during chronic hepatitis induces dysfunctional hyporesponsive T cells with increased expression of inhibitory molecules PD-1 and CTLA-4.^8^, ^29^, ^30^ We found that HBV-infected HIS-HUHEP mice had increased numbers of CD3^+^ T cells in the liver (low dose, 7x; high dose, 6x) and spleen compared with controls (Figure 4*A*). Following infection, CD4^+^ T_H_ cells were expanded in both the liver (5x for both low and high doses) and spleen. Furthermore, CD8^+^ T_C_ cell numbers increased massively in the liver (low dose, 9x; high dose, 6x), which is consistent with their role as key effectors in HBV clearance (Figure 4*B*). Strikingly, in the liver of high-dose inoculated mice, the majority of T cells shifted from a naïve (CD45RA^+^) to a memory phenotype (CD45RO^+^) (Figure 4*C*), resulting in the generation of an increased pool of effector memory cells (CD8^+^CD45RO^+^HLA-DR^+^) (Supplementary Figure 5). Liver-derived memory CD4^+^ and CD8^+^ cells in high-dose inoculated mice showed an exhausted phenotype with increased expression of PD-1 (Figure 4*D*). Interestingly, the frequency of PD-1^+^ memory CD4^+^CD45RO^+^ T cells correlated to viral loads in the liver of low-dose inoculated mice (Figure 4*E*).

**Supplementary Figure 6:**
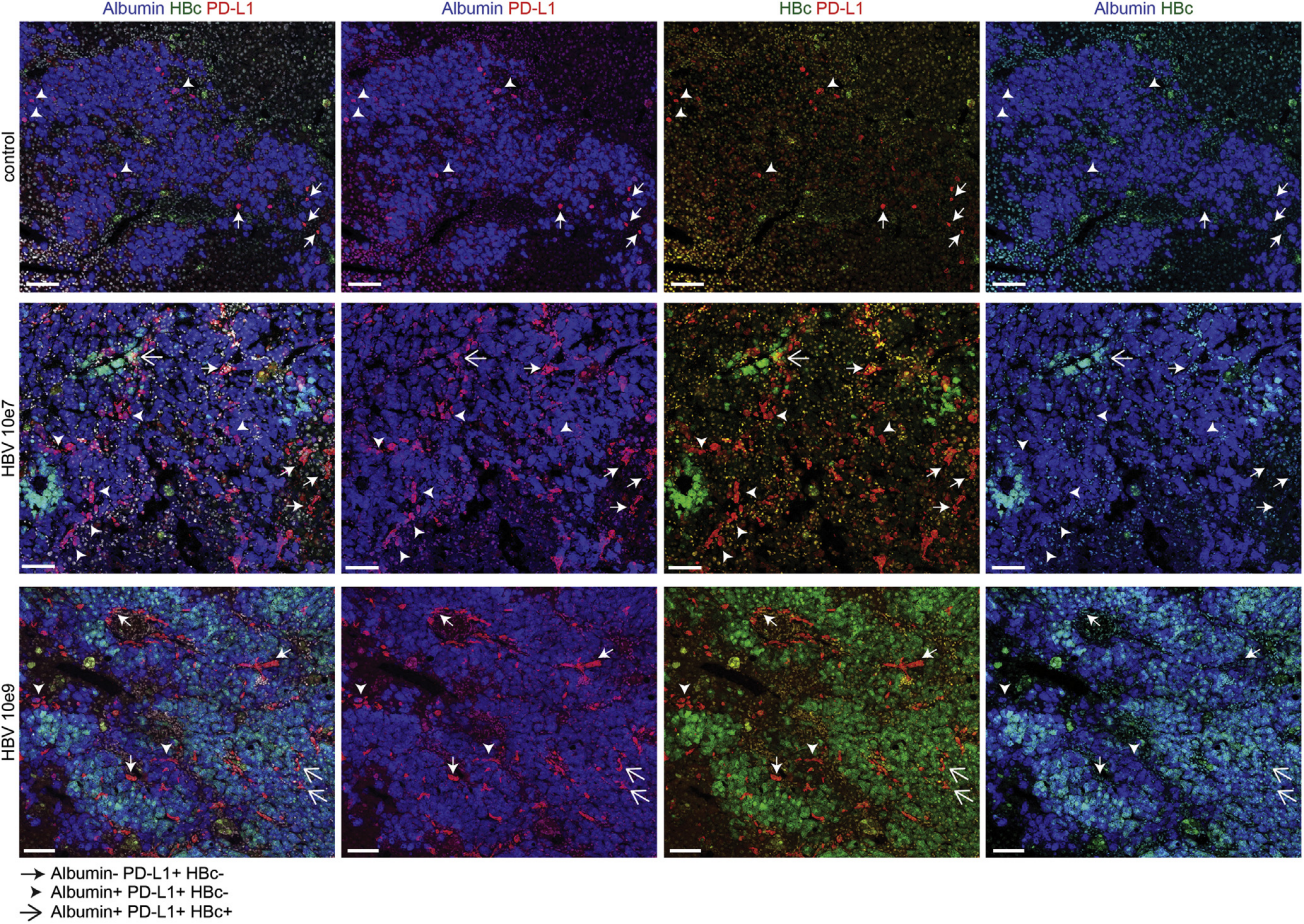
Immunofluorescence analysis of liver sections from HIS-HUHEP control or HBV-inoculated mice at either low dose (HBV 10e7) or high dose (HBV 10e9) co-stained for hAlbumin (*blue*), HBc (*green*), and PD-L1 (*red*) with DAPI-stained nuclei shown in *grey*. Scale bar represents 100 μm. In the second, third, and fourth vertical panels 1 color channel has been removed to visualize the staining patterns by pairs. *White arrowheads*: double-stained cells for Albumin and PD-L1; *white open arrows*: triple-stained cells for Albumin, HBc, and PD-L1; *white filled arrows*: cells mono-stained cells for PD-L1.

We next analyzed PD-1 ligand (PD-L1) expression in the liver of HIS-HUHEP mice. In non-infected mice, PD-L1 was expressed by few human hepatocytes (hAlb+) as well as non-parenchymal cells (hAlb-) (Supplementary Figure 6). During chronic HBV infection, PD-L1^+^ cells were far more abundant and distributed among immune cells as well as infected and non-infected hepatocytes (HBcAg^+^ or HBcAg^-^) (Supplementary Figure 6). These results suggest that in the context of chronic HBV infection, enhanced expression of PD-L1 by hepatocytes and non-parenchymal cells may engage PD-1 on T cells, thereby contributing to their exhaustion.

### Antigen-Specific Antibody Responses in HBV-Infected HIS-HUHEP Mice

**Figure 5:**
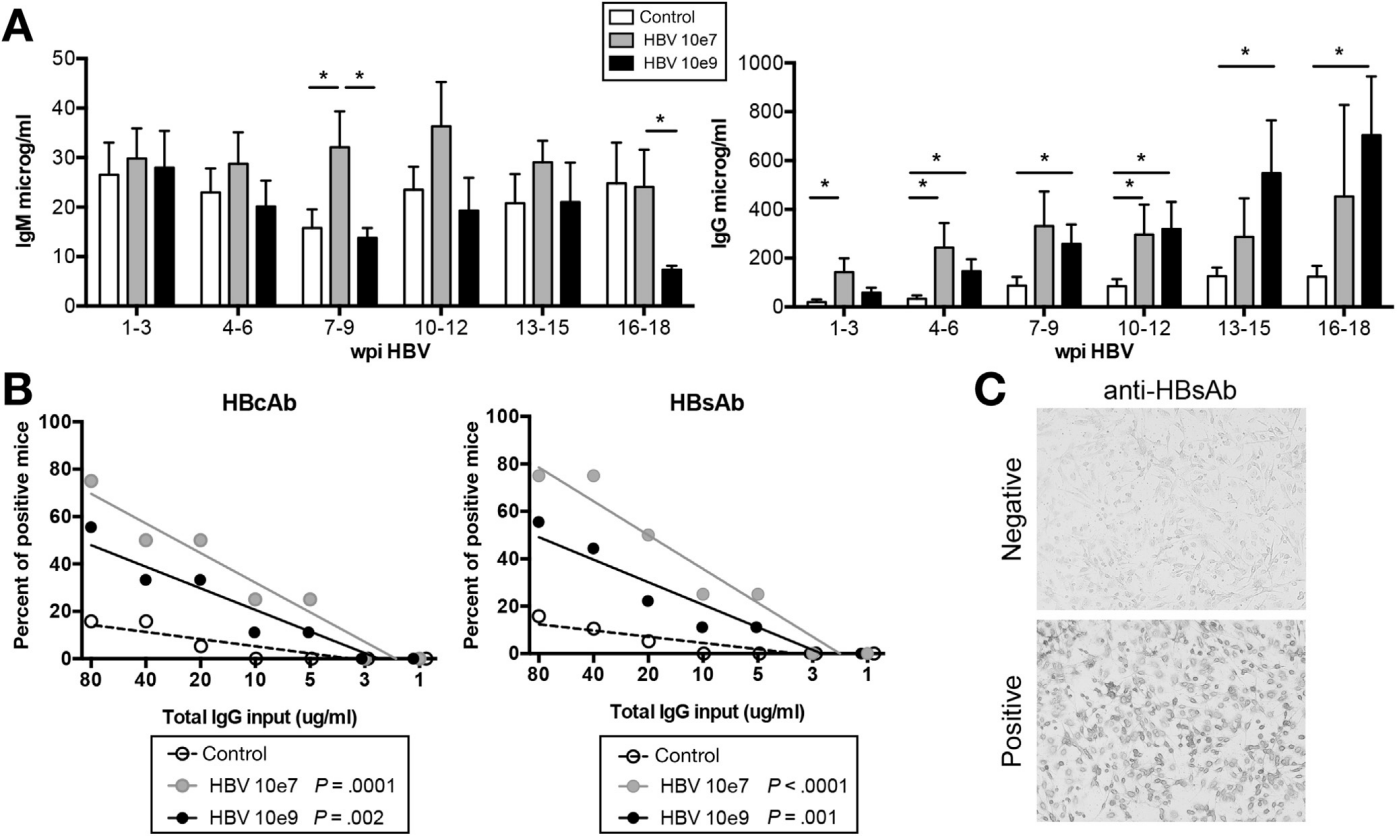
Humoral immune responses in HBV-infected HIS-HUHEP mice. (*A*) Total human IgM and IgG concentrations in the plasma of HIS-HUHEP control (*white bar*; n=11) or HBV-infected mice (10e7 inoculum: *grey bar*; n=5, 10e9 inoculum: *black bar*, n=9) plotted against weeks post infection (wpi). Bars show the mean with SEM. Statistics used unpaired two-tailed t test. (*B*) Analysis of anti-HBV antibodies in HIS-HUHEP mice. The frequency of mice positive for HBcAb IgG or HBsAb IgG from serially diluted plasma at the indicated concentration of total human IgG was analyzed by nonlinear regression. HIS-HUHEP control (n=19, *dotted line*), HBV-inoculated at 10e7 (n=4, *grey line*) or 10e9 (n=9, *black line*). *P* values indicate the differences between the slopes compared with the control data. (*C*) Representative images of IHC for anti-HBsAb.

Absolute numbers of splenic B cells (CD19^+^CD20^+^) were unchanged after ‘low dose’ or ‘high dose’ HBV infection (data not shown). Total hIgM levels were similar for control and ‘high dose’ infected mice, whereas in the ‘low dose’ inoculated mice antibody titers increased at 7–9 week post-infection (wpi) (Figure 5*A*). Class-switched hIgG antibodies increased significantly in both the ‘low dose’ and ‘high dose’ infected animals compared with controls, with an accelerated maturation in the ‘low dose’ inoculated mice (Figure 5*A*). We assessed anti-HBs (HBsAb) and anti-HBc (HBcAb) specific IgG antibody responses using a previously validated, highly sensitive immunohistochemistry assay.^24^ Both HBsAb and HBcAb responses were detected in the plasma in a majority of HBV-infected mice (low dose, 75%, n=4; high dose, 55%, n=9) (Figure 5*B* and 5*C*). HBV-specific antibody responses developed in a slightly higher fraction in ‘low dose’ vs ‘high dose’ infected mice. In line with previous reports,^31^ polyreactive IgGs could be detected in a few control HIS mice (16%, n=19). These results indicate that HIS-HUHEP mice generate HBV-specific IgG antibodies in response to infection.

### Human Biomarker Disease Profiles for Chronic HBV in Humanized Mice

**Figure 6:**
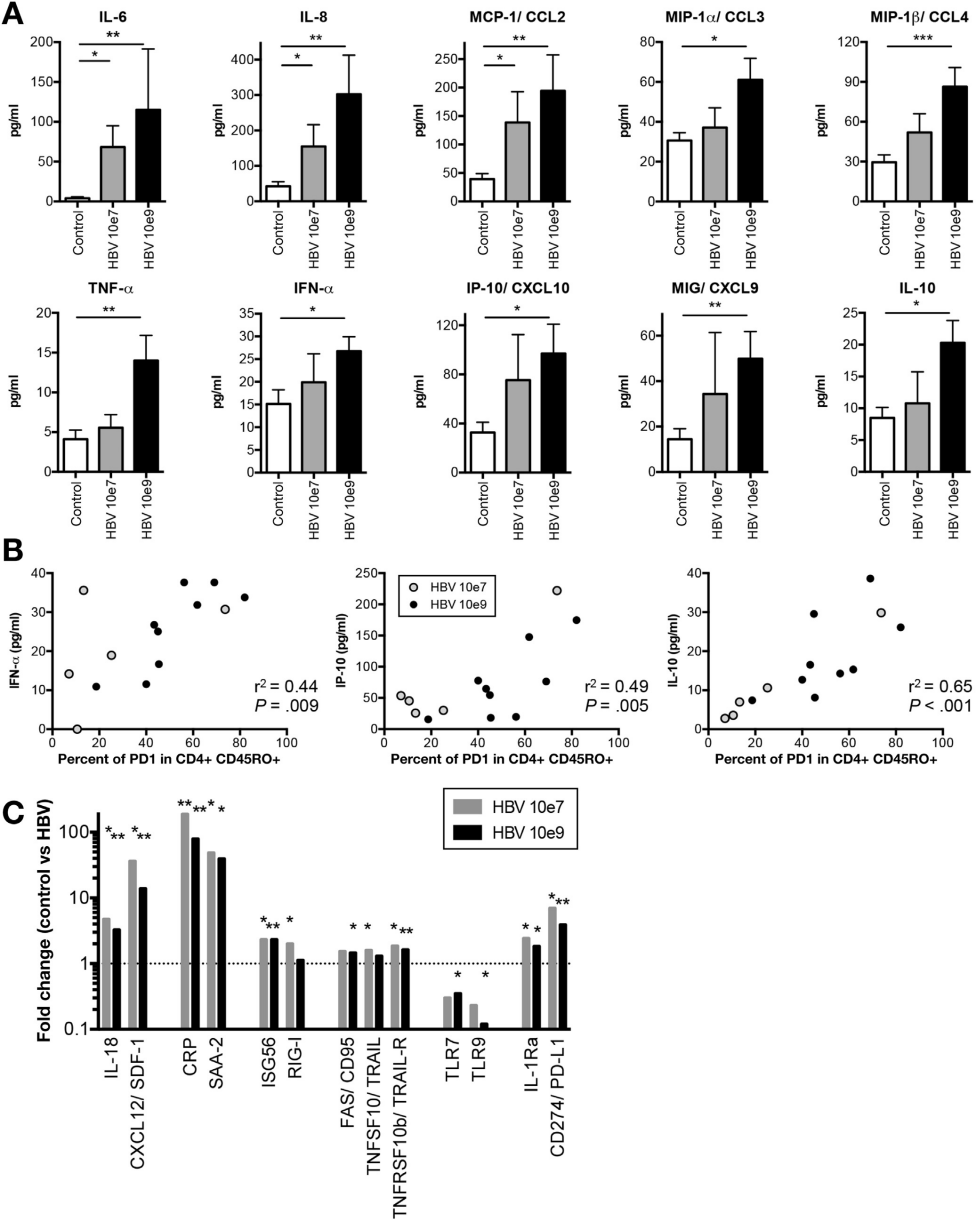
Biomarker analysis in HBV-infected HIS-HUHEP mice. (*A*) Plasma from HIS-HUHEP control (n=11) and HBV-infected mice (inoculum 10e7, n=5; or 10e9, n=10) at endpoint were analyzed using human cytokine multiplex assay. No cross-reactivity with mouse cytokines was detected. Plasma levels of IL-1β, IL-1Ra, IL-2, IL-2R, IL-4, IL-12, IL-13, IL-15, IFN-γ, GM-CSF, and RANTES were similar between control and HBV-infected mice; while IL-5, IL-17, and Eotaxin were not detected (data not shown). (*B*) Correlation of PD-1^+^ memory CD4^+^ CD45RO^+^ T cells with either IFN-γ, or IP-10/CXCL10, or IL-10 plasma cytokines quantified in (*A*). Each dot represents a mouse: grey or black inoculated with, respectively, HBV 10e7 or HBV 10e9. (*C*) RT-qPCR analysis of liver samples from HIS-HUHEP control (n=14) and HBV-infected mice (inoculum 10e7, n=5; or 10e9, n=9). Fold changes in gene expression of HBV-infected compared with control mice are shown. Data was normalized to the internal control human GAPDH (hGAPDH) to account for differences in humanization levels on triplicate samples. *Dotted line* indicates fold change of 1. Histograms show the mean and SEM. Data from 14–20 wpi. Statistical significance: Mann Whitney U tests.

**Supplementary Figure 7:**
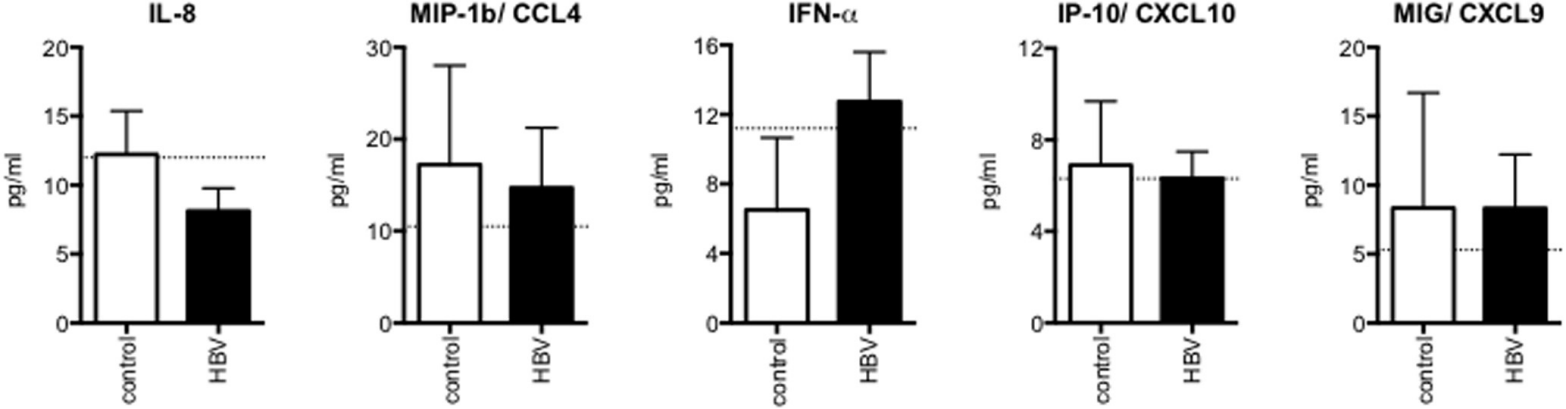
Biomarker expression patterns in HBV-infected and control HUHEP mice. Plasma from control (n=4) and HBV-infected (n=10) mice at the time of sacrifice (20 ±2 wpi) was analyzed by multiplex assay for human cytokines. IL-1b, IL-2, IL-2R, IL-4, IL-5, IL-6, IL-10, IL-12, IL-13, IL-15, IL-17, CCL2, CCL3, CCL5, GM-CSF, IFN-γ, and TNF-α were not detected. *Dotted lines* indicate the lower limit of quantification for each assay.

Although HBV is considered a “stealth” virus, modest elevations in cytokine and chemokine levels are observed in HBV-infected patients during the acute phase or hepatic flares.^32^, ^33^, ^34^, ^35^ To identify serum biomarkers in HBV-infected mice, plasma was screened by multi-analyte profiling. In non-infected HUHEP mice, very low levels of IL-8, CCL4, IFN-α, CXCL9, and CXCL10 could be detected that did not significantly increase following HBV infection (Supplementary Figure 7). Similarly, in non-infected HIS-HUHEP mice, IL-1b, IL-12, IL-1Ra, IL-2R, CCL5, and IFN-γ, could be detected but did not significantly change post-infection. In contrast, HBV-infected HIS-HUHEP mice had significantly increased levels of inflammatory mediators IL-6, IL-8, CCL2, CCL3, CCL4, TNF-α, as well as IFN-α and the interferon stimulated genes CXCL9 and CXCL10 (Figure 6*A*). Furthermore, the immunosuppressive cytokine IL-10 was also upregulated (Figure 6*A*). Biomarker profiles from low-dose inoculated mice had a moderate inflammatory signature, with significant increases observed only in IL-6, IL-8, and CCL2. Interestingly, plasma levels of CXCL10, IL-10, and to a lesser extent IFN-α, correlated with the expression levels of PD-1 on memory CD4^+^ T cells (Figure 6*B*).

We further analyzed gene expression profiles in the liver. Expressions of pro-inflammatory molecules CCL3, CCL4, IL-8, IL-18, CXCL9, CXCL12, and of acute phase response genes IL-6, C-reactive protein, and serum amyloid A2, were up-regulated in HBV-infected HIS-HUHEP mice (Figure 6*C* and data not shown). Although a weak IFN-α response was detected in the plasma of low-dose infected HIS-HUHEP mice, a clear increase in interferon-stimulated genes ISG56, and RIG-I was observed in the livers (Figure 6*C*). Previous studies showed that NK and T cells eliminate HBV-infected hepatocytes via Fas (CD95) and TRAIL-mediated apoptosis.^32^, ^36^ In the liver of HIS-HUHEP infected mice, we also observed increased expression of Fas/CD95, TRAIL, and TRAIL-R (Figure 6*C*). HBV antigens act as immunosuppressors by inhibiting the activity of innate sensors Toll-like receptor (TLR) 7 and 9 that recognize viral nucleic acids.^37^, ^38^ Similarly, TLR7 and TLR9 expression were downregulated in HIS-HUHEP–infected livers (Figure 6*C*). Moreover, immunosuppressive mediators (IL-1Ra and PD-L1) were also overexpressed in HBV-infected mouse liver (Figure 6*C*). In HUHEP livers following HBV infection, IL-18 and CXCL-12 were not detected and C-reactive protein, SAA-2, ISG56, RIG-I, FAS, TRAIL, TRAIL-R, TLR7, TLR9, IL-1Ra, and PD-L1 were not significantly modulated (data not shown).

### HBV-Infected Liver Progenitor Cells

**Supplementary Figure 8:**
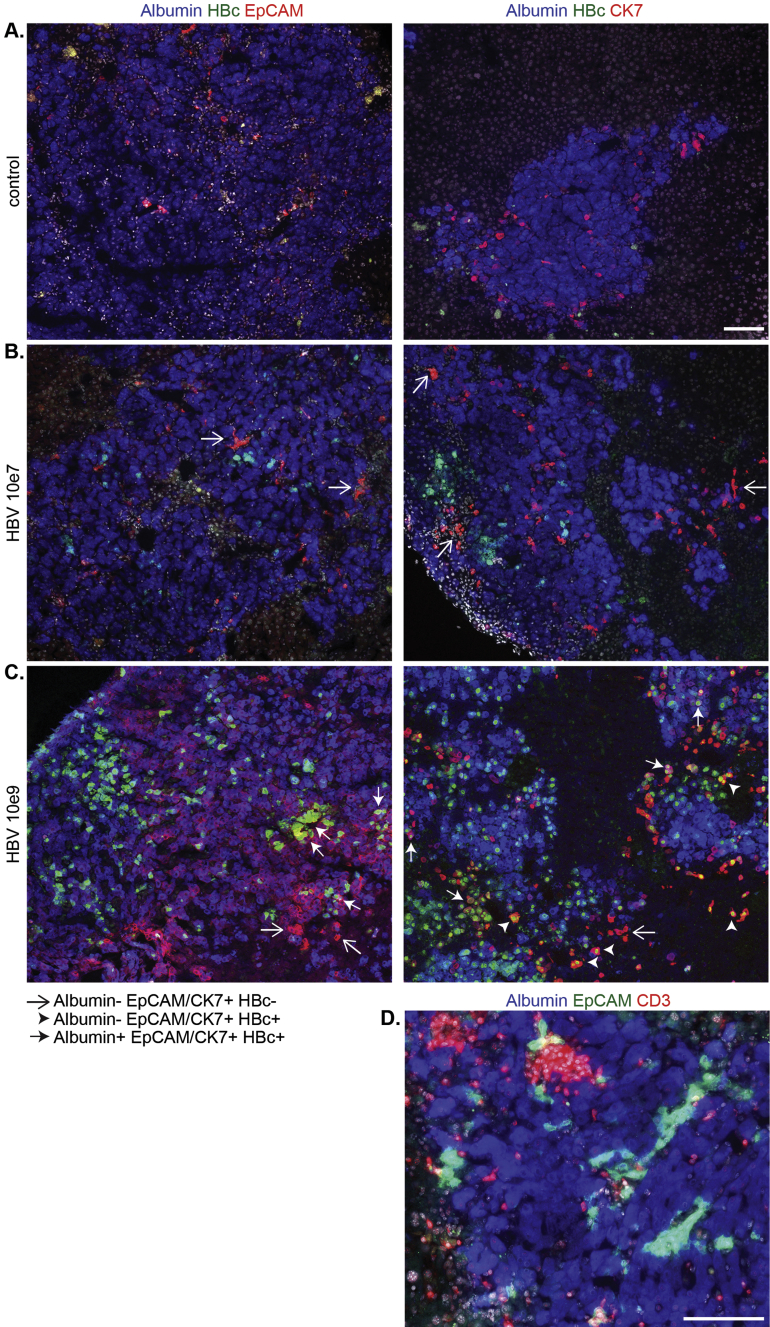
HBV-infected liver progenitor cells in highly viremic HIS-HUHEP mice. Immunofluorescence analysis of liver sections from (*A*) control, or HBV-infected mice inoculated with (*B*) HBV 10e7 or (*C*) HBV 10e9 for Albumin (*blue*), HBcAg (*green*) and either (*left column*) EpCAM (*red*) or (*right column*) CK7 (*red*). *White open arrows* show mono-stained cells for either EpCAM or CK7; *white arrowheads* show double-stained cells co-expressing HBc and CK7; *white filled arrows* show triple-stained cells co-expressing Albumin, HBc, and either EpCAM or CK7. (*D*) HBV-infected liver co-stained for Albumin (*blue*), EpCAM (*green*) and CD3 (*red*). Scale bar represents 100 μm.

Inflammatory cytokines (IL-6, TNF-α, IFN-γ) produced by macrophages or activated T and NK cells have been shown to induce proliferation of liver-progenitor cells (LPC) in response to liver damage.^22^, ^39^, ^40^ The more aggressive HCC subtypes express LPC markers (EpCAM, CK7, CK19, CD133)^41^, ^42^ and increased incidences of HCC have been correlated to high viral loads in CHB patients.^9^ EpCAM^+^Alb^+^ and CK7^+^Alb^+^ LPCs were observed in non-infected HIS-HUHEP mice, as expected in the context of liver regeneration because of continuous expression of the uPA transgene (Supplementary Figure 8*A*).^43^ Interestingly, in HBV-infected mice, some LPC were HBc^+^ and the more dedifferentiated progenitor-like CK7^+^Albumin^**-**^HBc^+^ cells were only observed in the high-dose infected mice (Supplementary Figure 8*B* and 8*C*). These HBc^+^ LPCs could originate from infected hepatocytes that had dedifferentiated, or may have been infected after their dedifferentiation to LPCs. Some LPCs localized near CD3^+^ T cell clusters, suggesting inflammation may impact on their development (Supplementary Figure 8*D*).^44^

### Testing Anti-Viral Therapies in HBV-Infected HIS-HUHEP Mice

**Figure 7:**
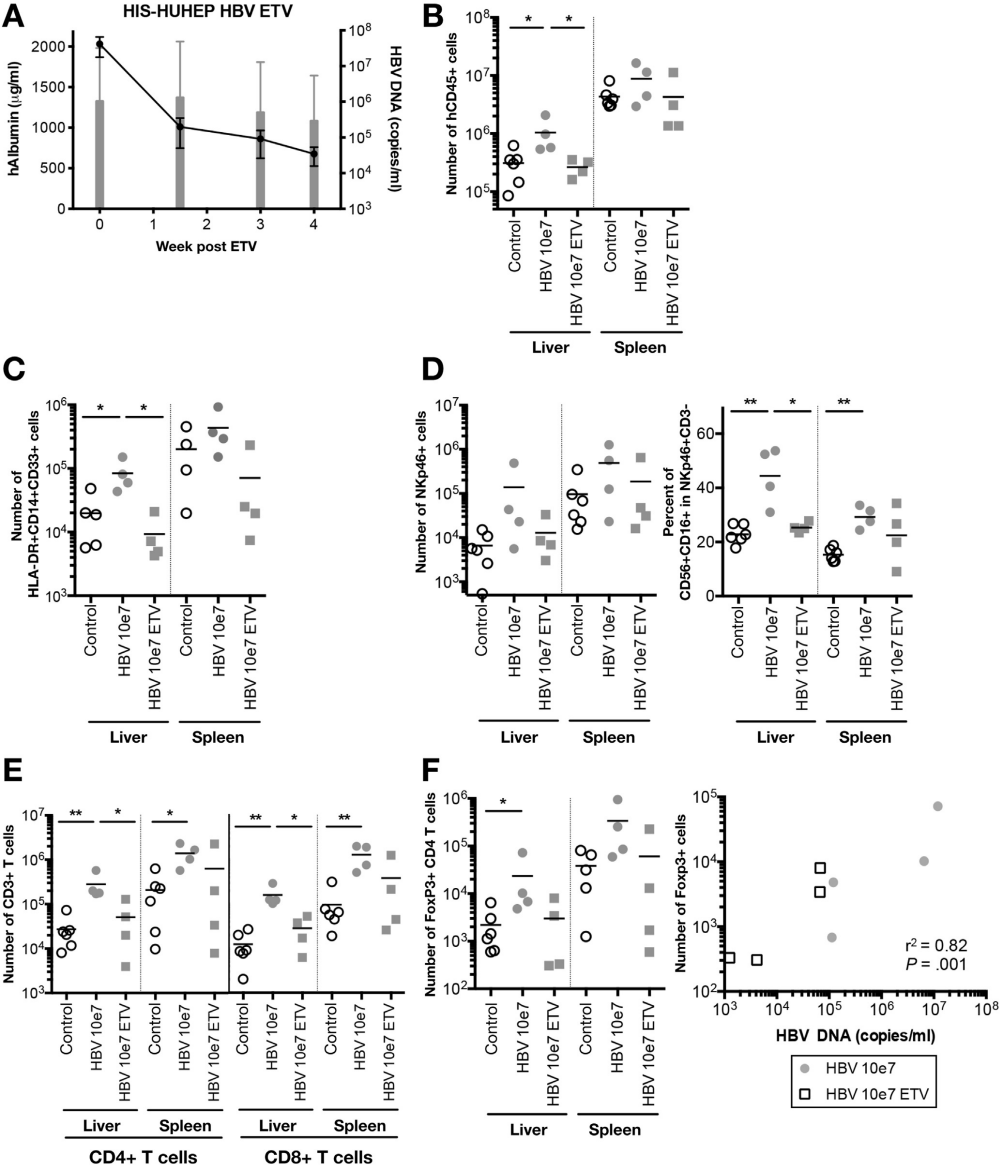
ETV treatment of HBV chronically infected HIS-HUHEP mice reverses hepatitis. (*A*) Liver engraftment (hAlbumin: *grey bars*) and viremia (HBV DNA: *black line*) in the plasma of mice previously infected for 3 months undergoing ETV treatment (n=4 mice). (*B* to *F*) FACS analysis of intrahepatic leukocytes and splenocytes from control, HBV-infected, and HBV-infected post-ETV treated mice. Absolute numbers of (*B*) total human leukocytes (hCD45^+^), (*C*) monocytes (HLA-DR^+^CD14^+^CD33^+^), (*D*) NK cells (CD3^-^NKp46^+^), and the frequency of CD56^+^CD16^+^ cells among NK cells, (*E*) CD4+, CD8+ (*F*), and Foxp3+ T cells. The number of intrahepatic Foxp3+ T_REG_ cells was plotted against the viral load at end point for each mouse and analyzed with Pearson’s correlation test.

**Supplementary Figure 9:**
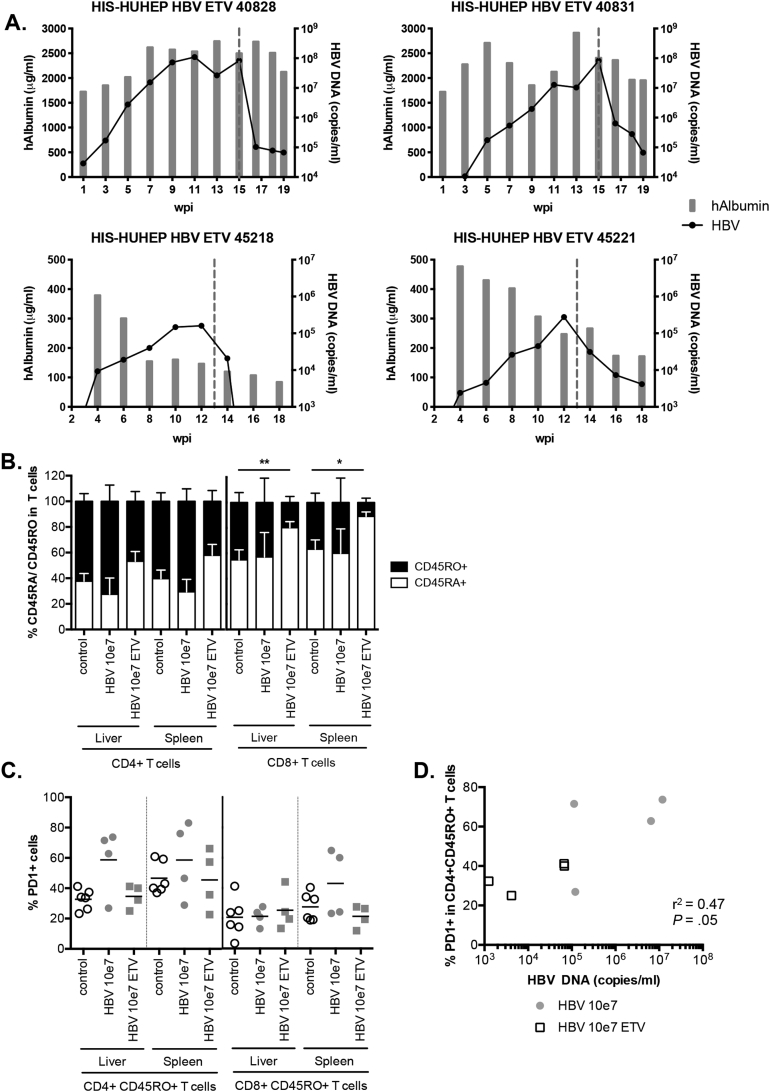
(*A*) Longitudinal analysis of viremia and liver chimerism in HBV-infected (10e7) ETV-treated HIS-HUHEP mice. The *dotted line* indicates start of treatment. (*B*–*D*) FACS analysis of T-cell phenotypes in control, HBV-infected (10e7), and HBV-infected (10e7) ETV-treated, HIS-HUHEP mice. (*A*) The frequency of naïve (CD45RA^+^) vs memory (CD45RO^+^) CD4^+^ and CD8^+^ T cells was evaluated in the liver and spleen of each cohort. (*B*) The frequency of PD-1^+^ cells in memory (CD45RO^+^) CD4^+^ or CD8^+^ T cells from the liver and spleen. (*C*) Correlation analysis of the viral load (HBV DNA) relative to the frequency of memory CD4^+^CD45RO^+^PD-1^+^ T cells in the liver using Pearson’s correlation test.

To determine whether suppression of viremia modifies intrahepatic immunophenotypes, we treated chronically HBV-infected HIS-HUHEP mice with the nucleoside analogue entecavir (ETV). ETV efficiently reduced viral loads (> 3 logs) in HBV-infected mice; viremia was undetectable in 2 of 4 treated mice (Figure 7*A* and Supplementary Figure 9). ETV-treated mice showed decreased liver inflammation with reduced monocytes and NK cells, including pro-inflammatory NK cells (CD56^+^CD16^+^), at levels comparable with noninfected mice (Figure 7*B*, *C,* and *D*). CD4^+^ and CD8^+^ T cellularity diminished and returned to a more naïve phenotype (CD45RA^+^), while the frequency of PD-1^+^ memory CD4^+^CD45RO^+^ cells tended to decrease (Figure 7*E* and Supplementary Figure 9). Among immunoregulatory mechanisms induced by HBV infection, T_REG_ accumulation in the liver of CHB patients dampens the antiviral immune response.^8^ Interestingly, whereas HBV-infected HIS-HUHEP mice had more T_REG_ CD4^+^CD25^+^Foxp3^+^ cells in the liver than controls, they were reduced after ETV treatment, and T_REG_ cellularity correlated with viral loads (Figure 7*F*). Thus, ETV treatment resulted in decreased viral loads and restored naïve immune phenotypes in the liver.

## Discussion

HIS-HUHEP mice constitute a robust small animal model to investigate bi-directional interactions between the immune system and the liver during hepatotropic infections by HBV. The strength of this model relies on the combination of stable hepatic chimerism that supports high levels of HBV replication, coupled with multilineage development of myeloid and lymphoid cell subsets recruited to the liver following infection. Although HIS and HUHEP grafts are not HLA matched, non-infected HIS-HUHEP mice showed no signs of graft vs graft responses. No inflammation or hepatotoxicity was observed, most likely because of the timing of grafts and the tolerant liver environment.^18^ Nevertheless, HBV-infected HIS-HUHEP mice developed chronic hepatitis that persisted for several months with viral titers, HBe and HBs antigenemia, cccDNA, and anti-viral immune reactions that were similar to those observed in CHB patients. Inoculation with lower or higher doses in HIS-HUHEP mice resulted in viremia similar to patient samples categorized as moderate vs elevated.^45^ Taken together, HIS-HUHEP mice represent a versatile system in which the molecular and cellular mediators of human antiviral immune responses can be studied. In this report, we investigated how viral loads affected the quality of the anti-HBV immune response and the outcome of viral infections.

The cellular distribution and phenotypes of immune mediators in the liver are distinct from those found in the periphery, thus analysis at the site of infection is key to deciphering anti-viral immunity.^4^ In HBV-infected HIS-HUHEP mice, Kupffer cells swarmed the liver, while NK- and T-cell recruitment increased proportionally to the dose of viral inoculum. Chemokine attraction has been suggested to play a role in this process via CXCL9 and CXCL10/CXCR3 interactions during hepatic flares in patients.^35^ We found that hepatic MIP-1α, MIP-1β, CXCL9, and CXCL10 levels were increased in chronically infected mice, which could participate in continuous monocyte, NK-cell, and T-cell recruitment to the liver. Intrahepatic NK cells were activated (CD56^+^CD16^+^CD69^+^) and functionally producing IFN-γ and TNF-α ex vivo following re-stimulation. The role of NK cells in mediating viral clearance vs persistence is still unresolved: they are essential in killing virally infected cells, yet they can eliminate HBV-specific T cells via TRAIL-mediated pathways, thereby contributing to pathogen maintenance.^7^, ^46^, ^47^ Liver-resident CXCR6+ NK cells have elevated TRAIL expression that may play a role in modulating T-cell–dependent HBV immunity.^48^ Because NK- and T-cell recruitment were positively correlated with viral infection in HIS-HUHEP mice, it seems unlikely that NK cells were eliminating hepatic HBV-reactive T cells in this context. Still, HIS-HUHEP mice should offer a useful tool to further study intrahepatic NK-cell responses during different phases of HBV infection where NK cells may modulate anti-viral responses.

A broad and robust T-cell response is essential for viral clearance, yet in the context of CHB patients, excess stimulation by viral antigens induces hypo-responsive PD-1^+^ T cells.^8^, ^11^, ^30^ We found that PD-1^+^ T cells were similarly enriched in the livers of HBV-infected HIS-HUHEP mice. Infected hepatocytes and antigen-presenting cells constituted a source of PD-L1 expression that may create an immunosuppressive environment and promote PD-1 mediated T-cell exhaustion. Interestingly, in low-dose inoculated and ETV-treated mice, the frequency of PD-1^+^ memory CD4^+^ T cells correlated with viral loads. This result suggests that low viral loads can be effectively controlled via anti-viral T cells up to a threshold level. Once this viral load is exceeded, T-cell responses may become ineffective, in part through PD-1 induction. Accordingly, in high-dose infected HIS-HUHEP mice, excessive viral production may overwhelm T-cell responses with uniform conversion to PD-1^+^ phenotype. While PD-1 blockade can improve anti-HBV T-cell responses in vitro,^11^, ^30^ this does not occur in all patients. Other co-inhibitory receptors, including CTLA-4, Tim-3, LAG-3, and 2B4, may have non-redundant roles in regulating HBV-specific T-cell responses.^29^

CD3^+^ T cells were specifically recruited to HBV-infected human hepatocyte foci (but not uninfected human hepatocytes), suggesting that human T cells are activated in a virus-specific fashion. However, because of the HLA-mismatched setting of these HIS-HUHEP mice (and eventually PD-1–dependent exhaustion), we were unable to detect HLA-specific T-cell responses (data not shown), which could have added to the usefulness of the model. Future studies in HLA transgenic and haplotype-matched immune system/hepatocyte HIS-HUHEP mice may help to address this issue. Still, antigens from virally infected hepatocytes may be cross presented by dendritic cells via MHC class I to CD8^+^ T cells, and in chronic HBV, CD14^+^ monocyte-derived dendritic cells carry HBV antigens to induce CD8^+^ T-cell responses.^49^ These mechanisms may allow HBV proteins to gain access to the MHC class I pathway to generate virus-specific T-cell responses in our model.

The balance between immunosuppressive and stimulatory signals regulates immune cell recruitment and activation in the liver of CHB patients and contributes to the pathophysiology during hepatic flares. This interplay was mirrored in HBV-infected HIS-HUHEP mice that had elevated pro-inflammatory mediators along with immunoregulatory cytokines in the plasma and T_REG_ recruitment to the liver. Plasmatic IL-10, CXCL10, and IFN-α levels correlated with the frequency of intrahepatic PD-1^+^ memory CD4^+^ T cells, which also correlated to HBV viremia, suggesting that the viral antigen load is able to fine tune the immune response via multiple pathways. These secreted biomarkers could facilitate the non-invasive evaluation of intrahepatic immune responses.

Antiviral treatments with NUC analogs control viral replication yet do not deplete cccDNA pools and rarely lead to functional cures. However, long-term treatments lead to improved clinical outcomes with reversion of hepatitis, fibrosis, and cirrhosis in CHB patients. HBV-specific polyfunctional T-cell responses and quiescent NK-cell phenotypes are partially restored in peripheral blood mononuclear cells from treated patients, yet the mechanisms regulating viral-host responses following NUC treatments are unclear.^47^, ^50^ ETV treatment of HBV-infected HIS-HUHEP mice reduced viral loads and restored naïve immune profiles, with diminished liver infiltration of monocytes, inflammatory NK cells, CD4^+^ and CD8^+^ T and T_REG_ cells. These results suggest that HBV viral loads are sensitively detected by the immune system and demonstrate the proof-of-concept utility of HIS-HUHEP mice for evaluating therapeutic strategies.

Because of the lack of robust prognostic biomarkers of HCC development and the invasiveness of repeated biopsy sampling in human liver, the evolution of HBV-related pathology to HCC development is still obscure. HCC tumor samples from CHB patients express elevated levels of LPC gene transcripts (*EpCAM*, *CK19*, *AFP*) and these LPCs are tumorigenic in immunodeficient NOD/SCID mice, suggesting that they may constitute tumor-initiating cells.^42^, ^51^ Hepatic ectopic lymphoid structures have been associated to LPCs with poor HCC prognosis.^44^ Following 4 months of high viremia and chronic inflammation in HIS-HUHEP livers, HBV-infected LPCs were observed in inflammatory sites, although HCC did not develop in our model. The human timeframe for HCC development (several decades) would be difficult to match in a mouse model. Nevertheless, chronically infected HIS-HUHEP mice may help to define tumorigenic pathways that are relevant to HCC initiation and progression.

The lack of effective therapeutic strategies to eliminate HBV, or induce a sustained anti-viral immune response off treatment, remains a major health problem. Adults co-infected with HIV and HBV have a higher incidence of liver-related mortality, although the underlying mechanisms remain obscure. Our HIS-HUHEP model could offer a means to understand disease pathophysiology in this context. Taken together, humanized immune system and liver mouse models provide a potential platform to test innovative therapeutic anti-HBV strategies that combine direct-acting antivirals with immunomodulatory drugs to assess and eventually harness the full potential of the human innate and adaptive immune response to cure HBV infections.

## Supplementary Materials and Methods

### Generation of Humanized Mouse Model

BALB/c *Rag2*^-/-^*Il2rg*^-/-^*Sirpa*^NOD^uPA^tg/tg^ male and female newborn mice (<1 week old) were sublethally irradiated (2.5 Gy), injected intrahepatically with ∼2x10^5^ CD34^+^ human fetal liver cells (Advanced Bioscience Resources, Alameda, CA), and subsequently injected intrasplenically with 7x10^5^ freshly thawed human hepatocytes (BD, Corning, Corning, NY) at 5–8 weeks. HUHEP mice were injected only with human hepatocytes as described above.

### Leukocyte Stimulation In Vitro and Intracellular Staining

For ex vivo cell stimulations, splenocytes (depleted of mouse CD45 cells with microbeads, Miltenyi Biotech) and intrahepatic lymphocytes were cultured overnight in RPMI 10% fetal calf serum penicillin streptomycin, with or without IL-12 (5 ng/mL), IL-15 (5 ng/mL), and IL-18 (20 ng/mL). GolgiPlug (BD Biosciences) was added 3 hours prior to cell staining with antibodies. IFN-γ, TNF-α (Miltenyi Biotech) and Foxp3 (eBioscience) intracellular labeling was performed after staining for extracellular proteins, fixation, and permeabilization of cells using either BD Cytofix/Cytoperm reagents (BD Bioscience) for IFN-γ and TNF-α, or Foxp3 Transcription factor Fixation/Permeabilization kit (eBioscience), according to manufacturer’s instructions.

### Analysis of Anti-HBs and Anti-HBc Antibody Responses

Human plasma samples were initially titrated with the Abbott Architect i2000SR analyzer (Chicago, IL). BHK-21 cells were transfected by electroporation with RNA transcribed in vitro from either pSFV1-SHBs^adw^ or pSFV1-HBc^adw^ or pSFV3-β-Gal constructs.^24^ Sixteen hours after transfection, cells were fixed in 80% acetone for 10 minutes at -20°C, and immediately used for immunohistochemical staining. Cells were incubated for 30 minutes at 37°C with serial 2-fold dilutions of humanized mouse plasma or patient serum first normalized to 80 μg/mL total immunoglobulin G (IgG). After 4 phosphate-buffered saline (PBS) washes, cells were incubated for 30 minutes at 37°C with a peroxidase-conjugated goat anti-human IgG antibody Fc gamma fragment specific (cat #109-035-008; Jackson ImmunoResearch Laboratories, West Grove, PA) diluted 1:500 in PBS. Cells were washed with PBS (4 times) and stained with an AEC (3-Amino-9-ethylcarbazole) substrate-chromogen solution (Sigma-Aldrich, Saint Louis, MI) for 5 minutes. After a wash with distilled water, wells containing red stained BHK-21 cells were scored using an Olympus IX51 microscope. The experimenter was not informed of the plasma's origin so as to ensure a blind interpretation of the results.
